# Draft Genome Sequence of *Mortierella alpina* Isolate CDC-B6842

**DOI:** 10.1128/genomeA.01180-13

**Published:** 2014-01-23

**Authors:** Kizee A. Etienne, Marcus C. Chibucos, Qi Su, Joshua Orvis, Sean Daugherty, Sandra Ott, Naomi A. Sengamalay, Claire M. Fraser, Shawn R. Lockhart, Vincent M. Bruno

**Affiliations:** a Centers for Disease Control and Prevention, Atlanta, Georgia, USA; b Institute for Genome Sciences, University of Maryland School of Medicine, Baltimore, Maryland, USA; c Department of Microbiology and Immunology, University of Maryland School of Medicine, Baltimore, Maryland, USA

## Abstract

We report the draft genome sequence of *Mortierella alpina* isolate CDC-B6842. *M. alpina* is a nonpathogenic member of the *Mucoromycotina* subphylum of fungi that is an important model for understanding the molecular mechanisms of lipid production and metabolism.

## GENOME ANNOUNCEMENT

Fungi belonging to the subphylum *Mucoromycotina* consist of a diverse group of organisms with industrial, biotechnological, and clinical relevance. They have been studied for their use in commercial applications, such as with biodiesel and other lipid-based products (1, 2). *Mortierella alpina*, a saprophytic member of *Mucoromycotina*, has been considered an important oleaginous model organism for understanding lipid metabolism and production. Unlike other genera of *Mucoromycotina*, *Mortierella* spp. are not known to cause disease in plants, animals, or humans. Sequencing the genomes of several isolates from this diverse group of fungi will allow us to understand the evolution of pathogenicity among the fungi within this subphylum.

*M. alpina* strain CDC-B6842 was isolated from the skin of a human leg lesion in Minnesota in 2004 but was demonstrated to be nonpathogenic. Genomic DNA was extracted using the GeneRite kit (Carlsberg, CA), according to the manufacturer’s instructions. The genomic DNA was sequenced at the University of Maryland School of Medicine, Institute for Genome Sciences, Genomics Resource Center (http://www.igs.umaryland.edu) using a combination of paired-end libraries (average insert of 483 bp) and mate-pair (3-kb) libraries on the Illumina HiSeq 2000. A total of 44.5 million 100-bp reads were generated. The draft genome data were assembled using the MaSuRCA genome assembler (3). The resulting genome assembly contained 1,185 contigs with an average size of 33,358 bp. This resulted in a predicted genome size of 39.53 Mb with a G+C content of 50.4%. Both the estimated genome size and G+C content are consistent with those calculated for *M. alpina* strain ATCC 32222 (2).

Structural and functional predictions were done using the Institute for Genome Sciences (IGS) eukaryotic annotation pipeline protocol 1.0 at the Institute for Genome Sciences, Informatics Resource Center (http://www.igs.umaryland.edu). Briefly, repeat annotation was performed using RepeatModeler (http://www.repeatmasker.org) and RepeatMasker (4). Genes were predicted *ab initio* using four gene prediction programs: CEGMA (5), GeneMark-ES (6), Augustus (7), and SNAP (8). Augustus and SNAP used CEGMA predictions for parameter training. Spliced alignments of Swiss-Prot protein models against the *M. alpina* genome were generated with AAT (9), using cutoffs of 60% identity and 80% similarity. All structural evidence was combined using EVidenceModeler (10). A total of 9,666 protein-coding genes, 233 tRNA genes, and 10 rRNA genes were predicted from this pipeline. While our estimation of the number of tRNA genes is consistent with what was reported for strain ATCC 32222, we estimate fewer protein-coding genes (2).

### Nucleotide sequence accession numbers.

This whole-genome shotgun project has been deposited at DDBJ/EMBL/GenBank under the accession no. AZCI00000000. The version described in this paper is the first version, AZCI01000000.
